# Raman Analysis of Orientation and Crystallinity in High *T*_g_, Low Crystallinity Electrospun Fibers

**DOI:** 10.1177/00037028231202791

**Published:** 2023-09-29

**Authors:** Arnaud W. Laramée, Christian Pellerin

**Affiliations:** 1 Département de chimie, 5622 Université de Montréal, Montréal, QC, Canada

**Keywords:** Anisotropic, applications, materials, polymers, quantitative analysis, Raman spectroscopy

## Abstract

Electrospun fibers of amorphous or low-crystallinity polymers typically exhibit a low molecular orientation that can hamper their properties and application. A key stage of the electrospinning process that could be harnessed to mitigate the loss of orientation is jet rigidification, which relates closely to the solvent evaporation rate. Here, we establish quantitative Raman methods to assess the molecular orientation and crystallinity of weakly crystalline poly(2,6-dimethyl-1,4-phenylene oxide) fibers with varying diameters. Our findings demonstrate that solvent volatility can be leveraged to modulate the orientation and crystallinity through its impact on the effective glass transition temperature (*T*_g,eff_) of the polymer jet during the electrospinning process. Specifically, a highly volatile solvent yields a higher and more sustained orientation (median ⟨*P*_2_⟩ of 0.53 for diameters < 1.0 µm) because its fast evaporation rapidly increases *T*_g,eff_ above room temperature. This vitrification early along the jet path promotes the formation of an oriented amorphous phase and a moderate fraction of strain-induced crystals. Our data reveals that a high *T*_g_ is a crucial parameter for reaching high orientation in amorphous or low-crystallinity polymer systems.

## Graphical abstract

**Graphical Abstract:**
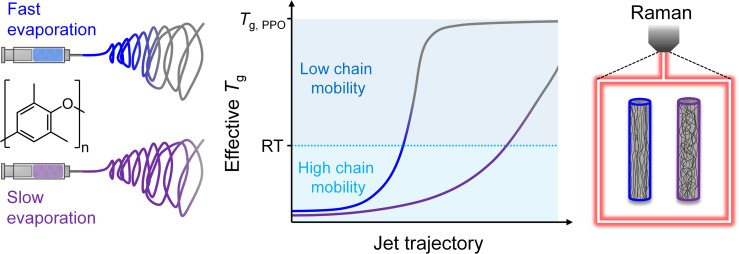
This is a visual representation of the abstract.

## Introduction

Electrospun fibers spark considerable interest due to their appealing mechanical,^1–8^ thermal,^9–11^ optical,^12^ and electrical^13,14^ properties, which tend to improve markedly at submicron diameters. This distinctive feature has been correlated with a drastic increase in molecular orientation (i.e., the alignment of polymer chains along the fiber axis) in the finest fibers.^4–7,15–17^ Vibrational spectroscopy is a powerful tool to establish the processing–structure relationships in these materials, notably to identify key parameters to optimize their performance in various fields such as coatings, filtration, tissue engineering, electronics, and energy harvesting.^18,19^

The impact of several electrospinning parameters such as the nature of the polymer^20^ and its molecular weight,^21^ the applied voltage,^22^ the distribution of the electric field along the polymer jet,^23–25^ the spinneret–collector distance,^26^ and the type of collector^27–29^ used to retrieve the fibers, has been studied at the mat level by infrared (IR) spectroscopy and X-ray diffraction. These works provided valuable insight into how the orientation of the mat can be modulated to target specific properties. However, results on collections of fibers are affected by their macroscopic alignment, their diameter polydispersity, and the presence of various types of defects such as beads and branches.^15,30^ Studies at the mat level thus provide imprecise information on the change of orientation as a function of fiber diameter and specific experimental parameters.

Some characterization techniques can alleviate these limitations by interrogating individual fibers rather than mats of fibers, most notably polarized confocal Raman microscopy,^16,17,31–35^ selected area electron diffraction,^36–41^ and IR photothermal-induced resonance techniques with atomic force microscopy or optical detection.^42^ Among these, Raman microscopy stands out because it allows faster and nondestructive quantification of molecular orientation in both crystalline and amorphous phases, thereby enabling a more efficient collection of diameter-dependent structural information.^15,30,31^ In particular, Raman studies on a series of increasingly crystalline polymers, i.e., polystyrene (PS),^16^ polyacrylonitrile (PAN),^17^ polyoxymethylene (POM), and poly(ethylene oxide) (PEO),^32^ revealed that orientation is higher and is maintained over a wider range of diameters for polymers with a higher crystallinity. This was explained by an increasing fraction of crystallites that act as effective crosslinking points and hinder chain relaxation.^32^ This conclusion was strengthened by our observation that highly crystalline PEO fibers show a high and nearly constant molecular orientation whether they are collected on a static collector or on a collector that provides additional mechanical or electric stretching.^34^ In contrast to PEO, fibers of low-crystallinity poly(ethylene terephthalate) (PET) show a much lower orientation that depends on the collector used.^33^ The formation of oriented crystalline or mesomorphous phases was monitored simultaneously by Raman and also depended on the type of collector, a result that was mainly attributed to the viscosity of the PET jet at the point where the collector induces additional stretching.^33^

This survey highlights that reaching a high orientation in fibers of amorphous or low-crystallinity polymers requires balancing the strain exerted on the jet during the electrospinning process and the mobility of the polymer chains. One way to control the mobility aspect is by choosing a solvent, or a mixture of solvents, whose volatility will dictate the viscosity of the polymer solution during fiber formation. While the electrospinning solvent is often chosen only for its ability to generate defect-free fibers, some studies have shown that higher volatility or the addition of a nonsolvent can promote phase separation in the jet, notably to prepare fibers with surface or internal pores.^43–46^ Other studies have established the impact of the solvent on the crystallization process and the formation of specific polymorphs.^47–49^ To our knowledge, the solvent has not yet been exploited as a tool to modulate the molecular orientation of amorphous and low-crystallinity fibers.

For such a study, a polymer with a high glass transition temperature (*T*_g_) is advantageous because it is more likely to retain a fibrillar morphology and exhibit measurable orientation in a solvent of lower volatility. We selected poly(2,6-dimethyl-1,4-phenylene oxide) (PPO) because it is a commercial polymer with a high *T*_g_ of ∼210 °C^50^ that can be processed into a low-crystallinity state.^51^ However, the Raman spectrum of PPO is not fully understood, and orientation-sensitive bands or quantitative indicators of crystallinity have not yet been clearly established. The determination of these spectral features is challenging but is necessary to probe the processing–structure–properties relationships of PPO.

This work thus has two primary purposes: (i) to identify and characterize orientation and structural markers in the Raman spectrum of PPO to expand its qualitative and quantitative exploitation, and (ii) to leverage this spectral information to establish a relationship between solvent volatility, molecular orientation, and crystallinity in individual PPO fibers of various diameters. Our Raman experiments, complemented by IR and differential scanning calorimetry (DSC) measurements, show that a highly volatile solvent results in the formation of fibers with a higher and more sustained level of orientation, primarily through the fast rigidification of the oriented amorphous chains into the glassy state.

## Experimental

### Materials and Electrospinning

Poly(2,6-dimethyl-1,4-phenylene oxide) (PPO) with a weight-average molecular weight of 50 kg/mol (Scientific Polymer Products, Cat. no. 126) was used as received. Chloroform (CHCl_3_) (≥ 99.8%, Fisher Chemical, Cat. no. C298500) and chlorobenzene (ClBz) (>98.0%, Tokyo Chemical Industry, Cat. no. C1948) were used as solvents without further purification. PPO fibers with diameters between 0.5 and 4 µm were prepared by dissolving an appropriate mass of PPO in 5 mL of solvent to obtain 14 and 18 wt% solutions in CHCl_3_ and 16 and 20 wt% solutions in a 50 : 50 v/v CHCl_3_/ClBz mixture. Once the PPO was fully dissolved (after about 3 h of magnetic agitation at room temperature [RT]), the solution was introduced in a 5 mL glass syringe fitted with a 0.41 mm diameter flat-end needle. A PHD 2000 syringe pump (Harvard Apparatus) was used to impose a constant flow of 0.03 mL/min. The fibers were spun in a fume hood at a relative humidity below 35% and temperatures between 20 °C and 22 °C. They were retrieved on a gap collector, with a 25 mm separation between the two metallic rods, located 15 cm from the needle tip. A 15 kV positive voltage was applied to the needle using a CZE 1000R high-voltage power supply (Spellman High Voltage Electronics) and a negative 2 kV potential was applied to the collector using a Power Designs source. The collection time was adjusted to yield sparse samples for the Raman experiments and denser samples for DSC analysis. The deposited fibers were transferred onto a microscope slide and dried under vacuum for at least 36 h before analysis to minimize residual solvent.

### Bulk Samples

Three types of bulk samples, referred to as *type A*, *type B*, and *type C*, were prepared to develop the quantitative spectroscopic methods.

#### Type A

Isotropic PPO films were cast from the 16 wt% PPO solution in CHCl_3_ into a Petri dish with a 90 mm diameter and left to dry at RT under a fume hood for 24 h. The film was then progressively heated up to *T*_g_, by increments of 20 °C or 30 °C (starting at 80 °C), in a vacuum oven over a 48 h period, and finally rapidly cooled down to RT under ambient conditions.

#### Type B

Poly(2,6-dimethyl-1,4-phenylene oxide) (PPO) samples of various crystallinity indices (*X*_c_) were prepared using the two following protocols. Samples of low crystallinity (*X*_c_ < 15%) were prepared by combining, in an aluminum DSC capsule, a few drops of the 16 wt% PPO solution in CHCl_3_ with 1–2 mg of the as-received PPO powder (*X*_c_ ∼ 32%). The mixture was homogenized in the DSC capsule using a thin glass rod. Samples of high crystallinity (15% < *X*_c_ < 30%) were obtained by adding one to two drops of CHCl_3_ to ∼1 g of PPO powder and recovering only the aggregated chunks resulting from the presence of solvent. If necessary, these chunks were subdivided into 3–4 mg samples before being deposited in an aluminum DSC capsule without subsequent homogenization. The solvent was evaporated by placing the unsealed capsules in a vacuum oven for 48 h and subsequently heating them around *T*_g_ for 30 min.

#### Type C

50 : 50 m/m binary films of PPO and atactic PS with a weight-averaged molecular weight of 942 kg/mol (Pressure Chemical) were prepared by evaporation of a 3% solution in CHCl_3_. The solution was deposited in a glass Petri dish with a diameter of 90 mm, previously placed on a heating plate preheated to approximately 55 °C. To avoid crystallization of PPO, a first evaporation stage was carried out without a cover for about 1 min. The Petri dish was then covered until the CHCl_3_ vapor started to condense, and then uncovered to enable rapid evaporation of the CHCl_3_ vapor built up above the film. The operation was repeated until most of the solvent was evaporated. These steps were carried out under a laminar flow cabinet to avoid the presence of dust in the film. The film was then placed under vacuum for at least 24 h at RT before being gradually heated 10 °C above the blend *T*_g_ for 48 h. The resulting film was detached from the Petri dish by capillarity by adding deionized water. The film was cut into 1 cm × 4 cm pieces with a thickness of about 40 µm. To induce different levels of molecular orientation, pieces were stretched to various draw ratios under heat gun heating at temperatures up to the blend *T*_g_. A DSC measurement performed on the film prior to its stretching revealed a single transition at 150 °C, corresponding to the expected *T*_g_ for a miscible 50 : 50 PS/PPO blend.

### Raman Spectroscopy

All Raman spectra were collected in the backscattering geometry using the 632.8 nm He−Ne laser of a LabRam HR800 spectrometer (Horiba Scientific) coupled with an Olympus BX41 microscope. For orientation quantification, the polarization of the incident laser beam and the Raman scattered light was set parallel (Z) or perpendicular (X) to the main axis (*z*) of the sample by using a half-wave plate and an analyzer, respectively. A 600 grooves/mm holographic grating was used with a scrambler to reduce the polarization dependence of its diffraction efficiency. Correction factors close to unity were applied to compensate for the residual polarization dependence of the instrument. Sets of parallel- and cross-polarized spectra were collected in the order *ZZ*, *ZX*, *XX*, *XZ*, *ZZ*(2), where the *ZZ*(2) spectrum was recorded to detect potential loss of focus or drift during acquisition. Any set of spectra was rejected when the intensity of the band used for orientation quantification differed by more than 5% in the *ZZ* and *ZZ*(2) spectra. The integration time, the number of accumulations, and other optical parameters were adjusted as specified in the following sections depending on the type of sample interrogated. The orientation parameter ⟨*P*_2_⟩ was calculated using the depolarization (depol) constant (DC) method described fully in previous work.^31^

#### Determination of the Tilt Angle

Polymer chain (*c*) orientation with respect to the *z*-direction is quantitatively represented by the ⟨*P*_2_⟩*_c_*_,*z*_ parameter, which takes a value of 0 for a completely isotropic system and a maximum value of 1 for the perfect orientation of the chains along the *z*-axis. However, Raman spectroscopy gives access to the ⟨*P*_2_⟩*_M_*_,*z*_ parameter that describes the orientation of the main axis of the Raman tensor of the mode considered (*M*) with respect to the *z*-axis. The ⟨*P*_2_⟩*_c_*_,*z*_ can be calculated from the experimental ⟨*P*_2_⟩*_M_*_,*z*_ by applying the Legendre addition theorem, provided that the average tilt angle between *M* and *c*, α*_M_*_,*c*_, is available:^52^

(1) $${\left\langle P_{2}\right\rangle }_{c,z}=\frac{2}{3\cos^{2}(\alpha_{M,c})-1}\cdot {\left\langle P_{2}\right\rangle }_{M,z}$$

Values of α*_M_*_,*c*_ are available in the literature for some polymers, such as PS and PET,^53–55^ but not for PPO to the best of our knowledge. Assuming that the Raman tensor has a cylindrical symmetry,^52^ Eq. 1 shows that a plot of ⟨*P*_2_⟩*_M_*_,*z*_ as a function of ⟨*P*_2_⟩*_c_*_,*z*_ results in a linear function whose slope is (3 cos2(α*_M_*_,*c*_) − 1)/2. The α*_M_*_,*c*_ angle was thus determined by measuring pairs of ⟨*P*_2_⟩*_M_*_,*z*_ (by Raman) and ⟨*P*_2_⟩ *_c_*_,*z*_ (by polarized IR) at precisely the same location for a series of type C samples covering a wide range of orientations*.* The details of the polarized IR measurements are provided in the Supplemental Material. For PPO, the *c*-axis is defined as the segment connecting the center of the first and third phenyl rings in a set of three consecutive repeating units adopting the most stable conformation, as initially introduced by Lefebvre et al.^56^

#### Determination of the Depolarization Ratio

The DC method requires knowledge of the depol ratio of the band of interest. As a value was not found in the literature, the depol ratio of the 1305 cm^–1^ band was determined by averaging the *ZX*/*ZZ* and *XZ*/*XX* intensity ratios measured at more than 10 locations across multiple isotropic samples (type A films and as-received powder). These spectra were recorded using a long working distance 100× objective (numerical aperture of 0.8). The confocal hole and the slit were set to 300 and 350 μm, respectively, the integration time was 3 s, and 10 accumulations were averaged.

#### Molecular Orientation in Individual Fibers

A small quantity of fibers was cut from the surface of the microscope slide with a scalpel, transferred with fine tweezers onto a BaF_2_ window, and immobilized with adhesive tape. Sets of polarized spectra were collected with an integration time of 6–8 s and six to eight accumulations on well-isolated individual fibers aligned with their main axis along the *Z* direction. The 100× objective was used with the confocal hole and slit set to 100 and 150 μm, respectively.

The diameter of each fiber was estimated by acquiring a linear map of 10 to 15 *ZZ* spectra at regularly spaced positions perpendicular to the fiber axis, with an integration time of 4 s and four accumulations. The spectral intensity was then plotted as a function of position and fitted to a Gaussian function using Origin software. The diameter was taken as double the computed standard deviation (σ) of the fit.

#### Crystallinity Quantification

Between three and eight sets of *ZZ* and *XX* spectra were recorded for each type B sample using a 10× objective. A higher number of spectra was recorded for more inhomogeneous samples to obtain a representative sampling. The integration time was 6–8 s and each spectrum was an average of 6–10 accumulations.

### Infrared Spectroscopy

Transflection measurements were carried on a Tensor 27 spectrometer (Bruker Optics) equipped with a HgCdTe detector cooled with liquid nitrogen to evaluate the relative evaporation rate of CHCl_3_ and ClBz in their 50 : 50 mixture. A fixed volume of solvent was deposited into a circular stainless-steel cup mounted on a Seagull (Harrick Scientific Products) variable angle reflection accessory with the incident and reflected angles set at 10°. The spectra were recorded as a time series with a resolution of 4 cm^–1^.

### Differential Scanning Calorimetry

The crystallinity of type B samples was investigated by DSC with a TA Instruments Q2000 calorimeter calibrated with indium. *X*_c_ values were determined from the first heating scan from 25 °C to 300 °C at a rate of 50 °C/min. The enthalpy of fusion of 100% crystalline PPO used to calculate *X*_c_ was set at 5.95 kJ/mol (49.5 J/g).^50^ The *X*_c_ value of type A samples was validated to be ∼0% using the same procedures.

### Multivariate Model for the Quantification of PPO Crystallinity From Raman Spectra

A multivariate calibration model was developed with TQ Analyst software to quantify crystallinity by relating the Raman of type A and B PPO samples to their crystalline and amorphous phase fractions determined by DSC. Samples were used either as calibration standards or as validation standards to test the model prior to its use for the quantification of fibers. To minimize the impact of orientation for oriented samples, “structural” Raman spectra (*I_S_*) were calculated from the *ZZ*- and *XX*-polarized spectra as:

(2) $$I_{s}=\frac{I_{ZZ}+2I_{XX}}{3}$$

The structural spectra obtained for a given sample were averaged to yield a representative sample spectrum. The sample spectra were then smoothed using a seven-point Savitzky–Golay filter to reduce noise.

The partial least squares (PLS) calibration technique was employed to build the model since it can adequately manage spectra in which component peaks overlap (as is often the case for peaks associated with crystalline and amorphous phases), baselines are variable, and peak shifting or broadening may occur.^57^ The variations in size and geometry of the samples (films, powders, and fibers) can induce optical distortions that were taken into account by introducing a standard normal variate (SNV) path length setting in the model. SNV is relevant when the effective optical volume of standards and unknown samples fluctuates and is challenging to determine accurately, when no band is solely proportional to the volume sampled, and when scattering or other optical effects cause spectral variations.^57^

## Results and Discussion

### Molecular Orientation

#### Band Selection and Characterization

**Figure 1.**
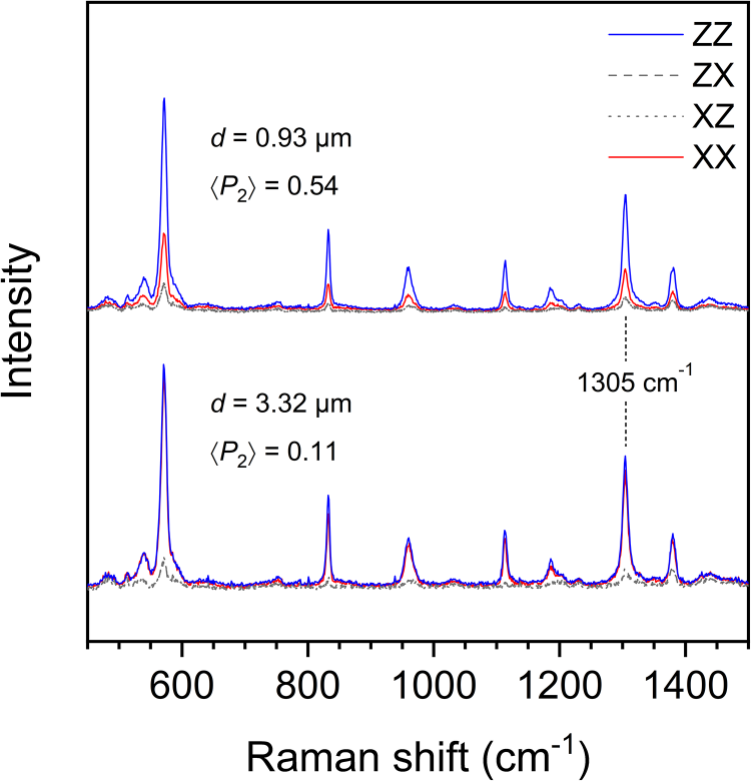
Sets of polarized Raman spectra collected on two representative PPO fibers of smaller and larger diameters.

PPO is a high-*T*_g_ semicrystalline polymer soluble in a breadth of organic solvents,^58^ making it an excellent material to define the impact of solvent volatility on the molecular orientation and structure in electrospun fibers. However, the incomplete knowledge of its Raman spectrum and the structural information it encloses calls for additional analysis to identify adequate quantitative markers. Figure 1 shows representative sets of polarized Raman spectra collected from two individual PPO fibers of small (top) and large (bottom) diameters (*d*), associated with high and low molecular orientation (represented by their order parameter ⟨*P*_2_⟩), respectively.

The polarization contrast strongly depends on fiber diameter for several bands, which indicates their sensitivity to molecular orientation. The bands at 572, 1305, and 1378 cm^–1^, associated with out-of-plane ring deformation, ring stretching, and –CH_3_ symmetrical deformation, respectively,^56,59,60^ show satisfactory intensity under both parallel- (*ZZ* and *XX*) and cross-polarized (*XZ* and *ZX*) polarizations, which helps to compute ⟨*P*_2_⟩ values confidently. The 1305 cm^–1^ band was preferred for orientation quantification because the position and width of this A_1_ mode remain constant upon orientation or crystallization, which strongly suggests that it is representative of the overall orientation of both amorphous and crystalline phases. Furthermore, it does not show significant overlap with other PPO bands (as does the most intense band at 572 cm^–1^) nor with bands of PS (as shown in Fig. S1, Supplemental Material), which allows us to use the mechanically advantageous PS/PPO blends to establish an orientation quantification method.

The intensity of the 1305 cm^–1^ band remains significant in the *XX* spectrum of the small fiber (Fig. 1), which suggests that the tilt angle of the Raman tensor differs from 0°.^56^ As shown in Eq. 1, a tilt angle different from 0° signifies that the ⟨*P*_2_⟩*_M_*_,*z*_ values computed from this band are not directly representative of the orientation of the polymer chain with respect to the fiber direction. Knowledge of the tilt angle of a vibration allows us to calculate the chain orientation ⟨*P*_2_⟩*_c_*_,*z*_ by correcting the as calculated ⟨*P*_2_⟩*_M_*_,*z*_ values, leading to a more intuitive and comparable orientation marker for polymer materials. Unknown Raman tilt angles can be determined by correlating the ⟨*P*_2_⟩*_M_*_,*z*_ Raman results with ⟨*P*_2_⟩*_c_*_,*z*_ values obtained from a complementary technique and the Legendre addition theorem,^52^ as detailed in the experimental section. In this context, polarized IR proved to be the complementary technique of choice because the IR spectrum of PPO is sufficiently well understood to derive ⟨*P*_2_⟩*_c_*_,*z*_ values. Specifically, the IR measurements were performed by polarized attenuated total reflection because it provides a sampling depth similar to that achieved with the Raman microscope. This is beneficial to the quality of the Raman–IR correlation because it avoids potential orientation inhomogeneities along the sample thickness. Transmission and specular reflection IR measurements were also considered, but the associated depth of analysis was either too large (transmission) or too small (specular reflection) to match that of the Raman measurements. Using this methodology (see Fig. S2, Supplemental Material), we found an average tilt angle of 33° for the 1305 cm^–1^ band. To validate our approach, we took advantage of the presence of PS in type C samples for which the Raman mode at 623 cm^–1^ has a reported tilt angle of 90°.^53,54^ The value determined experimentally is 89°, in excellent agreement with the literature. In the following sections, the orientation values reported for PPO are systematically corrected for the tilt angle and thus represent ⟨*P*_2_⟩*_c_*_,*z*_ values. For simplicity, they are referred to as ⟨*P*_2_⟩ values.

#### Impact of Solvent Volatility

**Figure 2.**
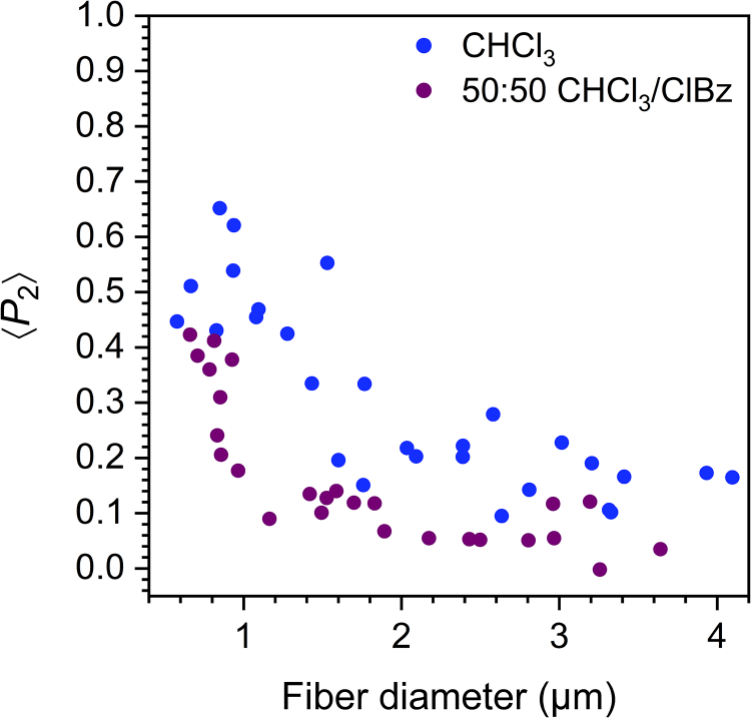
Diameter dependence of molecular orientation for PPO fibers electrospun using high-volatility CHCl_3_ and low-volatility 50 : 50 CHCl_3_/ClBz solvent systems.

Figure 2 illustrates the variation of molecular orientation as a function of fiber diameter for individually interrogated PPO fibers spun in either pure CHCl_3_ or a 50 : 50 CHCl_3_/ClBz blend. Both are good solvent systems in which PPO is completely solubilized with comparable ease.

The PPO fibers produced in pure CHCl_3_ stand out from typical amorphous or low-crystallinity fibers due to their remarkably high molecular orientation over a broad range of diameters. They also present an unusually large orientation onset diameter (*d*_0_) of ∼1.8 µm, defined as the diameter below which the orientation begins to increase more rapidly. More specifically, the median ⟨*P*_2_⟩ is 0.53 for small fibers of *d* < 1.0 µm and 0.18 for *d* > *d*_0_. By comparison, fibers of amorphous and low-crystallinity polymers spun under similar conditions typically exhibit a *d*_0_ inferior to 0.7 µm, a lower orientation at *d* < *d*_0_, and a negligible orientation at *d* > *d*_0_.^16,33^ This unusual behavior is likely due to the very high *T*_g_ of PPO (∼210 °C) and to the high volatility of CHCl_3_ (vapor pressure of 26.2 kPa at 25 °C^61^).

Theoretical and experimental works^62,63^ have highlighted that the strong elongational forces acting on the jet at a very high strain rate (on the order of 10^3^ s^–1^) are sufficient to induce almost complete polymer chain extension early in the electrospinning process, close to the jet starting point. At this stage, the effective *T*_g_ (*T*_g,eff_) of the PPO–CHCl_3_ system is well below RT because the solvent acts as a plasticizer in the jet. As a consequence, the competing chain relaxation is efficient (the relaxation time is small) such that this orientation only remains transient if it is not frozen in by the rigidification of the jet, either by crystallization or vitrification. The high *T*_g_ of pure PPO and the fast evaporation of CHCl_3_ combine to cause a rapid increase of *T*_g,eff_ and consequently a rapid increase of the chain relaxation time. Compared to a polymer of lower *T*_g_, the *T*_g,eff_ of PPO can rise above RT at an earlier stage of the electrospinning process, favoring the kinetic freezing of the jet in an oriented state. This phenomenon, from which the high orientation levels measured are believed to primarily arise, is exacerbated in thinner fibers because they are inherently subjected to larger stretching forces during their formation and because their higher surface-to-volume ratio favors an even faster solvent evaporation.^18,64,65^ Both factors contribute to a higher orientation in fibers of lower diameter by simultaneously increasing the strain rate and the relaxation time. The modeling of these two parameters in the jet, and how they evolve during solvent evaporation, would inform on whether orientation will be maintained or not in the resulting fibers. This is conceptually similar to the work by Pai et al.,^66^ where the characteristic times for fiber buckling and drying were modeled and allowed for predicting the final morphology of the fibers.

We note that an additional orientation may also occur as the system transitions into the vitreous state if the electrostatic forces acting on the solidifying charged jet remain momentarily strong enough to overcome its viscoelastic resistance to deformation. Finally, the collector may increase orientation by imposing an additional stretching in the solid state. This is most probable when using a drum collector rotating at a high linear velocity, as observed in our prior study on PET fibers.^33^ A gap collector was used in the present study to minimize this possible effect on the orientation results.

Consistent with this picture, PPO fibers produced from the 50 : 50 CHCl_3_/ClBz solvent mixture show lower orientation values than those spun from pure CHCl_3_ (Fig. 2), although we emphasize that their orientation is still higher than typically observed for low-crystallinity or amorphous polymers. The *d*_0_ associated with these fibers is ∼1.0 µm and the median ⟨*P*_2_⟩ values are 0.36 for *d* < *d*_0_ and 0.09 for *d* > *d*_0_. These decreases in orientation and *d*_0_ are essentially attributed to the much lower volatility of ClBz (vapor pressure of 1.6 kPa at 25 °C^61^) compared to CHCl_3_. Transflection IR measurements (see Fig. S3, Supplemental Material) show that it takes, for example, approximately three times longer to evaporate 90% of the initial CHCl_3_/ClBz mixture compared to pure CHCl_3_. The fraction of ClBz increases gradually during the evaporation process and it becomes essentially the only solvent still present when ∼22% of the initial solvent quantity remains. While the timescale of these solvent evaporation experiments is orders of magnitude longer than during the electrospinning process, because of the much larger surface-to-volume ratio of the electrospinning jet, we assume that the qualitative trends should apply in both cases. A rough estimation (see details below Fig. S3, Supplemental Material), based on the *T_g_* of the pure compounds and the changes in solvent composition during evaporation, suggests that achieving the *T*_g,eff_ = RT condition requires approximately 17% of CHCl_3_ or 22% of the CHCl_3_/ClBz mixture within the electrospinning jet. Although more solvent must evaporate to reach this condition in the case of pure CHCl_3_, Fig. S3 (Supplemental Material) shows that it occurs substantially later for the CHCl_3_/ClBz mixture due to the much lower vapor pressure of ClBz. This allows for more efficient chain relaxation in the region of the jet where the strain rate is highest and results in lower molecular orientation. In fact, the reduction of *d*_0_, from 1.8 µm to 1.0 µm, supports that larger elongational forces are required to compensate for the shorter relaxation time in the presence of a low-volatility solvent such as ClBz.

### Crystallinity

Crystallization during fiber formation can strongly impact molecular orientation, as shown in previous works on the molecular structure of fibers of various crystallinity.^17,32,33^ The presence of a crystalline phase in PPO has already been documented for bulk samples,^51^ but the ability of PPO to crystallize during electrospinning and its possible impact on the orientation process remain unknown. To our knowledge, quantitative markers of crystallinity have not been established in the Raman spectrum of PPO. This prompted the development of a method to quantify crystallinity at the individual fiber level.

#### Method Development

A two-component multivariate calibration model based on a PLS regression was constructed from a set of 20 PPO standards consisting of type A and B samples whose *X*_c_ (and proportion of amorphous phase, 1 – *X*_c_) was evaluated by DSC. The standards covered *X*_c_ values ranging from 0 to 32%, from fully amorphous films to as-received semicrystalline PPO powder. Each standard was characterized by Raman to obtain representative structural spectra prior to its DSC analysis. A first iteration of the model was built from the Raman spectra/*X*_c_ couples utilizing the entire spectral range and provided computed pure component spectra for the crystalline and amorphous phases. These spectra facilitated the identification of spectral markers for crystallinity with higher variance. Qualitative consideration of the experimental spectra of samples of lower and higher DSC crystallinity also guided the selection of spectral regions for the model.

**Figure 3.**
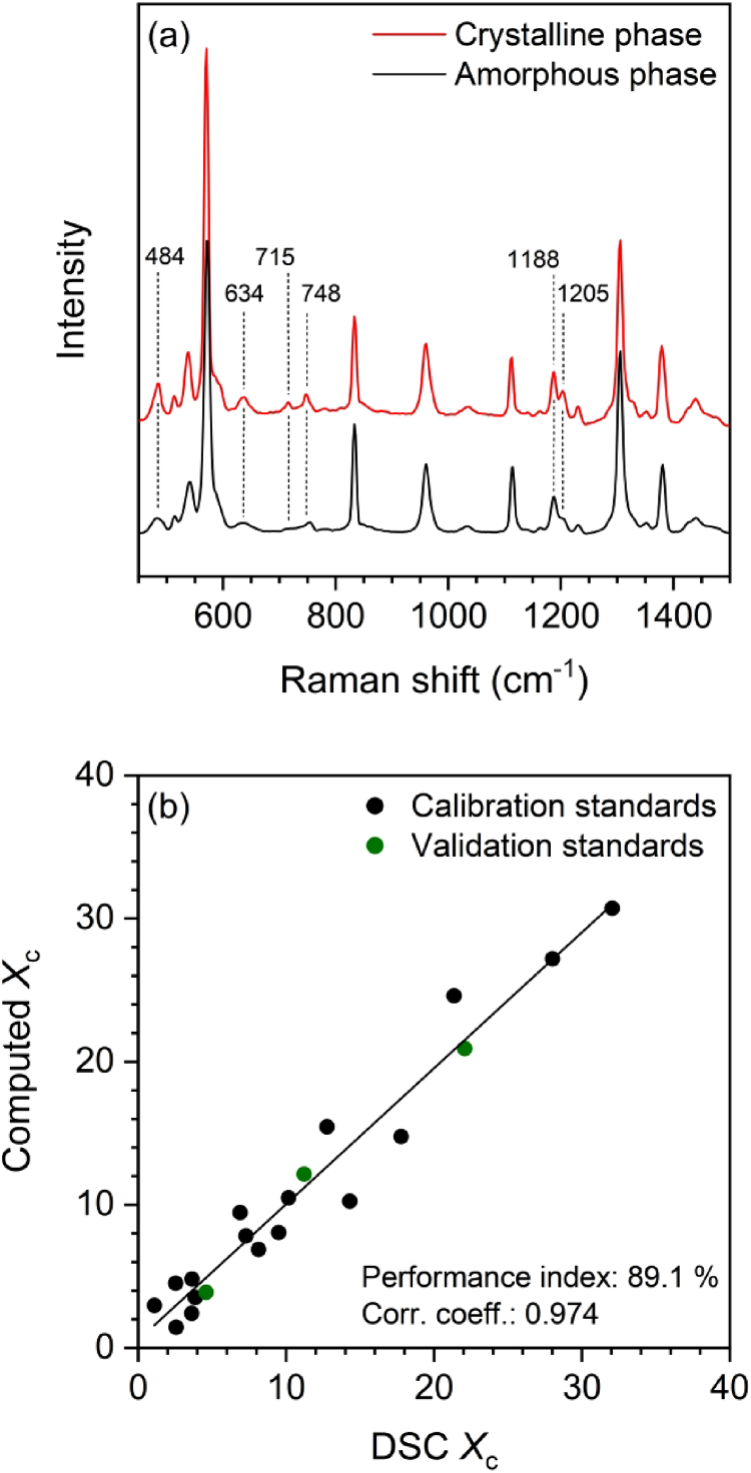
(a) Calculated pure component spectra for the crystalline and amorphous phases of PPO. (b) PLS calibration relating the crystallinity (*X*_c_) values of bulk PPO samples predicted from the Raman spectra to those determined by DSC.

Figure 3a shows the pure component spectra computed from the final version of the model. They are similar but display some distinctive features: the bands at 484, 634, 715, and 748 cm^–1^ are sharper in the crystalline phase spectrum, and the intensity ratio of the 1205 and 1188 cm^–1^ bands increases for samples of higher crystallinity. Interestingly, previous studies have reported some sensitivity to the degree of crystallinity for this region in the IR spectrum of PPO, but it has not been exploited quantitatively.^67,68^ Method diagnostics indicated the largest spectral variance (changes in band intensity, position, or width) associated with changes in *X*_c_ were present in the two spectral regions between 455 and 503 cm^–1^ and between 1170 and 1220 cm^–1^. Therefore, these two regions were utilized to build the model. On the other hand, the regions around the 634, 715, and 748 cm^–1^ bands were excluded due to their lower variance and modest signal-to-noise ratio, especially in the spectra of fibers. In parallel to the selection of the regions, a series of diagnostic tests were run iteratively to optimize the model,^57^ and qualitative checks validated that the calculated crystallinities were consistent with the appearance of the spectral markers for crystallinity for both the standards and the fibers.

Figure 3b shows the relation between the *X*_c_ values of the standards calculated from the Raman spectra (computed *X*_c_) and the DSC *X*_c_ values. The black and green symbols represent the calibration and validation standards, respectively. Four factors were needed to describe spectral variations as a function of *X*_c_ optimally. The correlation coefficient and performance index of the method are 0.974 and 89.1%, respectively, indicating good agreement between DSC and computed *X*_c_ values.^57^ The minimal fit quality values for all the fiber spectra quantified was 98.8% in the measurement regions considered. The uncertainties associated with the *X*_c_ values computed from the fiber spectra are all ≤4%, consistent with the root mean square error of cross-validation of 3.6% for the model.

#### Impact of Solvent Volatility

**Figure 4.**
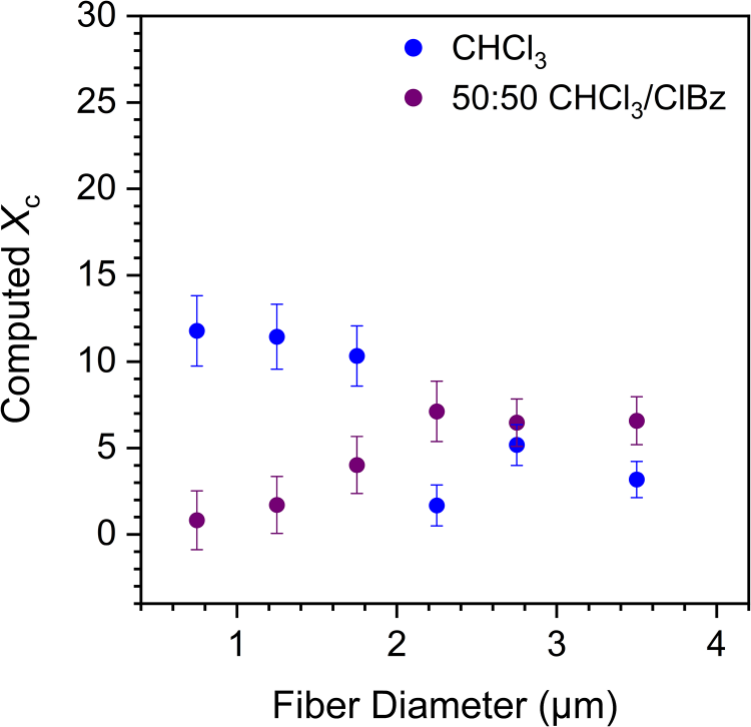
Diameter dependence of crystallinity for PPO fibers electrospun using high-volatility CHCl_3_ and low-volatility 50 : 50 CHCl_3_/ClBz solvent systems. The crystallinities were computed from the average of structural spectra within 500 nm diameter windows.

Figure 4 shows the *X*_c_ values of PPO fibers computed from the PLS model as a function of diameter. Each data point was obtained from the averaged structural spectra (obtained using Eq. 2) of the individual fibers within a given diameter range (0.5–1, 1–1.5, 1.5–2, 2–2.5, 2.5–3, or >3 µm) to improve the signal-to-noise ratio and the fit quality. The results confirm that the crystallinity of PPO in the fibers is globally low, with average *X*_c_ values around 7 and 5% for fibers produced in pure CHCl_3_ and the 50 : 50 CHCl_3_/ClBz mixture, respectively.

Fibers prepared in pure CHCl_3_ show a substantial crystallinity only at the smallest diameters, with *X*_c_ values centered at 11% for the three windows with *d* < 2.0 µm, in contrast with mean *X*_c_ values centered at 3% when *d* > 2.0 µm. The diameter below which crystallinity is higher matches well the onset diameter *d*_0_ of 1.8 µm noted for orientation (Fig. 2). This suggests that crystals developed under high strain in the thin fibers with *d* < *d*_0_. By contrast, fibers prepared in the 50 : 50 CHCl_3_/ClBz mixture show a small or negligible degree of crystallinity for thin fibers, with average *X*_c_ values as low as 2–3% at *d* < 2.0 µm, and an apparent increase of crystallinity as a function of diameter up to 7% at *d* > 2.0 µm. This suggests that crystals can also form quiescently when a larger fiber diameter and a low-volatility solvent combine to grant sufficient chain mobility under quasi-static conditions. An increasing degree of crystallinity in fibers of larger diameter was also reported for PAN fibers electrospun in dimethylformamide, also a low-volatility solvent.^8^

### Discussion

Our results indicate that the choice of solvent impacts both the molecular orientation and the crystallinity in PPO electrospun fibers, as well as their respective diameter dependences. Bearing in mind that previous studies have shown a correlation between these two structural characteristics in individual fibers,^32,33^ our objective here is to clarify the interplay between system rigidification and chain crystallization, and its incidence on fiber structure.

The high orientation of PPO fibers with *d* < *d*_0_ prepared in pure CHCl_3_ is mainly due to the *T*_g,eff_ > RT condition being reached quickly, which allows to kinetically freeze the oriented state generated by the high strain rate affecting the jet. It also stems, to a lesser extent, from the concomitant formation of strain-induced crystallites that can act as effective crosslinking points and further hinder the relaxation of neighboring oriented amorphous chains. At *d* > *d*_0_, the lower surface-to-volume ratio of the jet reduces the rate of solvent evaporation,^18,65^ resulting in a slower increase in *T*_g,eff_. This phenomenon, combined with the inherently smaller strain experienced by the larger fibers during their formation, limits the degree of orientation of larger fibers. The *X*_c_ values centered at 3% for *d* > 2.0 µm indicate that a modest crystallization can take place while the strain is low or absent, but it is not sufficient to explain the relatively high level of orientation preserved in fibers of large diameters compared to typical amorphous systems of lower *T*_g_. We conclude that the predominant factor for this orientation remains the achievement of the *T*_g,eff_ > RT condition.

**Figure 5.**
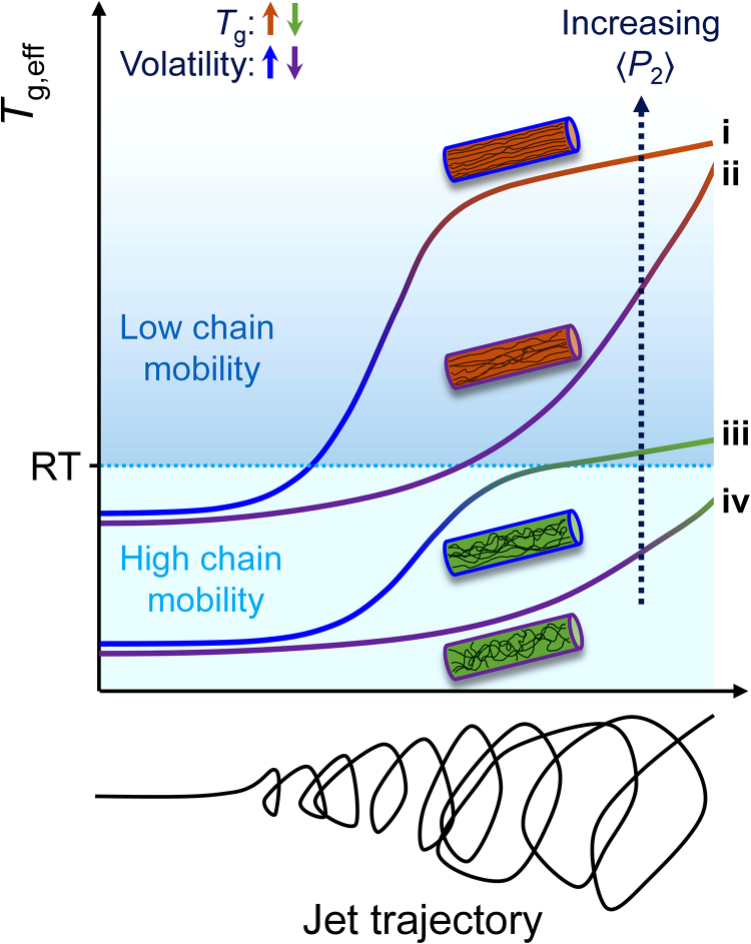
Schematic representation of the *T*_g,eff_ as a function of jet trajectory and the corresponding fiber orientation for (i) high-*T*_g_ polymer with high volatility solvent, (ii) high-*T*_g_ polymer with low volatility solvent, (iii) low-*T*_g_ polymer (*T*_g_ > RT) with high volatility solvent, and (iv) low-*T*_g_ polymer (*T*_g_ > RT) with low-volatility solvent. Vitrification occurs when *T*_g,eff_ exceeds RT. The progressive change in the color of the curves reflects the evolution of the jet composition toward the pure polymer due to solvent evaporation.

Following this rationale, the lower orientation found in PPO fibers of small diameters spun from the less volatile CHCl_3_/ClBz mixture is attributed to a lower *T*_g,eff_ in the jet compared to the CHCl_3_ counterparts (Fig. 5). This lower *T*_g,eff_ enables faster relaxation of the chain orientation and appears to impede stress-induced (oriented) crystallization in fibers with *d* < *d*_0_. Thanks to the high *T*_g_ of PPO, the *T*_g,eff_ > RT condition is still met during the electrospinning process for these fibers, leading to a substantial degree of molecular orientation in the amorphous phase. In contrast, larger fibers with *d* > *d*_0_ spun from the CHCl_3_/ClBz mixture present the lowest orientations despite substantial *X*_c_ values (up to 7% at *d* > 2.0 µm), confirming that mobility remained high enough throughout the formation of these fibers to relax most induced orientation.

Overall, the results point to rapid vitrification (*T*_g,eff_ > RT) as a critical requirement to obtain highly oriented fibers of amorphous or weakly crystalline polymers. An even lower volatility of the solvent would further increase the dominance of the relaxation processes over the strain-induced orientation and would likely lead to weakly oriented fibers over the complete diameter range, as typically observed for lower-*T*_g_ polymers even when they are electrospun from relatively high-volatility solvents.

Figure 5 provides a visual summary of the influence of the polymer *T*_g_ and solvent volatility on the system mobility, as schematically represented by the evolution of the *T*_g,eff_ values during the electrospinning process, for the four scenarios of a high-*T*_g_ polymer with a high or low volatility solvent, and a low-*T*_g_ polymer (with *T*_g_ > RT) with a high or volatility solvent. The case of a polymer with a subambient *T*_g_ is not represented but it should lead to vanishingly low orientation because, by definition, its *T*_g,eff_ cannot reach RT.

From this analysis, we establish that:

- To maximize the orientation in amorphous or low crystallinity electrospun fibers, it is crucial to choose a polymer with a high *T*_g_ and a highly volatile solvent;
- Gradually reducing the solvent volatility should allow producing fibers of a weakly crystalline polymer (with *T*_g_ > RT) to exhibit either (i) high orientation and strain-induced crystallization, (ii) moderately high orientation and minimal strain-induced crystallization, or (iii) low orientation and quiescent crystallization.

Interestingly, the requirements for high orientation in fibers of amorphous or low crystallinity polymers, such as PPO, contrast substantially with those associated with highly crystalline polymers, such as PEO and POM. These polymers can reach very high *X*_c_ values of 74 and 59%,^32^ respectively, but present a low *T*_g_ that is tens of degrees^69^ below RT such that vitrification never occurs. The high orientation in these fibers is explained by the ultrafast strain-oriented crystallization within the jet. In fact, the importance of meeting the condition *T*_g,eff_ > RT during the electrospinning process appears to be inversely related to the rate of crystallization of the electrospun polymer.

## Conclusion

This work showcases the development of quantitative Raman methods for the simultaneous evaluation of molecular orientation and crystallinity of PPO. These methods were applied to evaluate the impact of solvent volatility on the molecular structure and orientation of low-crystallinity PPO electrospun fibers of various diameters. We show that solvent volatility acts as a lever to tune the degrees of orientation and crystallinity via its effect on the effective glass transition temperature (*T*_g,eff_) during the electrospinning process. A highly volatile solvent leads to the highest and best-maintained orientation because vitrification (*T*_g,eff _> RT) occurs under conditions that favor the formation of both the oriented amorphous phase and strain-induced crystallization. Our data also reveal that a higher *T*_g_ is conducive to higher orientation, when all other parameters are kept constant, for amorphous or low-crystallinity polymers. This contrasts with the common perception that fibers of such polymer inevitably exhibit little anisotropy. This study finally illustrates how the development of quantitative Raman methods is key to elucidating the processing–structure–properties relationships of materials.

## Supplemental Material

sj-docx-1-asp-10.1177_00037028231202791 - Supplemental material for Raman Analysis of Orientation and Crystallinity in High *T*_g_, Low Crystallinity Electrospun Fibers Supplemental material, sj-docx-1-asp-10.1177_00037028231202791 for Raman Analysis of Orientation and Crystallinity in High *T*_g_, Low Crystallinity Electrospun Fibers by Arnaud W. Laramée and Christian Pellerin in Applied Spectroscopy
