# Supplementary material for: Raman Analysis of Orientation and Crystallinity in High Tg, Low Crystallinity Electrospun Fibers
Source: Appl Spectrosc. 2023 Sep 29;77(11):1289–99. doi: 10.1177/00037028231202791 (PMC10604433; doi:10.1177/00037028231202791)
Supplement: sj-docx-1-asp-10.1177_00037028231202791 - Supplemental material for Raman Analysis of Orientation and Crystallinity in High Tg, Low Crystallinity Electrospun Fibers [file sj-docx-1-asp-10.1177_00037028231202791.docx]

**Raman Analysis of Orientation and Crystallinity in High T_g_, Low Crystallinity Electrospun Fibers**

**Supporting Information**

Arnaud W. Laramée and Christian Pellerin*

*Département de chimie, Université de Montréal, Montréal, QC, H3C 3J7, Canada*

* Corresponding author: c.pellerin@umontreal.ca

*Selection of the orientation-sensitive Raman band*

**Figure S1.** Raman spectra of a PS film, a 50:50 PS/PPO film and a PPO fiber. The bands identified in the PPO fiber spectrum do not show significant overlap with the PS bands.

*Infrared characterization of the orientation-sensitive band*

Polarized attenuated total reflection (ATR) measurements on type C samples were performed at the same locations as by Raman using a Tensor 27 spectrometer (Bruker Optics) equipped with a HgCdTe detector cooled with liquid nitrogen and a Seagull ATR accessory (Harrick Scientific). The ATR element was a germanium hemisphere fitted on a Ming-Sung™ ATR sample rotator and the angle of incidence (θ) was 50°. The ⟨*P*_2_⟩_M(IR),z_ parameter, which describes the orientation of the transition dipole moment vector (M(IR)) with respect to the drawing (z) axis, was evaluated for the 1305 cm^-1^ (PPO) and 906 cm^-1^ (PS) bands from spectra with a resolution of 4 cm^-1^ polarized parallel (p) and perpendicular (s) to the plane of incidence. The z axis of the films was positioned perpendicular to the plane of incidence of light and the ⟨*P*_2_⟩_M(IR),z_ was calculated with the following adapted equations^1,2^:

$\left\langle P_{2} \right\rangle_{M\left( \mathrm{IR} \right),z}=(k_{z}-k_{y})/(k_{z}+{2k}_{y})$ (S1)

where *k_z_* and *k_y_* are the absorption indices parallel and perpendicular to the sample drawing direction, respectively. They were calculated from the experimental absorbances, *A*_s,z_ and *A*_p,z_, of the selected bands by assuming uniaxial orientation with cylindrical symmetry of the samples:

$A_{s,z}=K_{1}k_{z}$ (S2)

$A_{p,z}=K_{2}k_{y}+K_{3}k_{x}= k_{y}(K_{2}+K_{3})$ (assuming that $k_{x}$ *=* $k_{y}$) (S3)

where *k_x_* is the absorption index along the sample thickness and *K*_1_, *K*_2_, and *K*_3_ are constants that depend on the intensity of the electric field of the evanescent wave, the incidence angle θ, and the ratio of the refractive index of the sample and the ATR crystal (*n*_21_ *= n*_sample_*/n*_ATR_ *= 1.5/4.0*):

$K_{1}=\frac{4n_{21}^{2}cos\theta}{\left( 1-n_{21}^{2} \right)\left( {sin}^{2}\theta-n_{21}^{2} \right)^{1/2}}$ (S4)

$K_{2}=\frac{4n_{21}^{2}cos\theta({sin}^{2}\theta-n_{21}^{2})}{\left( {sin}^{2}\theta-n_{21}^{2}+n_{21}^{4}{cos}^{2}\theta\right)\left( {sin}^{2}\theta-n_{21}^{2} \right)^{1/2}}$ (S5)

$K_{3}=\frac{4n_{21}^{2}cos\theta{sin}^{2}\theta}{\left( {sin}^{2}\theta-n_{21}^{2}+n_{21}^{4}{cos}^{2}\theta\right)\left( {sin}^{2}\theta-n_{21}^{2} \right)^{1/2}}$ (S6)

Values of ⟨*P*_2_⟩_M(IR),z_ can be converted into the corresponding ⟨*P*_2_⟩_c,z_ using the Legendre theorem^3^:

$\left\langle P_{2} \right\rangle_{c,z}=\frac{2}{3{cos}^{2}(\alpha_{M(IR),c})-1}\cdot\left\langle P_{2} \right\rangle_{M\left( \mathrm{IR} \right),z}$ (S7)

thanks to the availability in the literature of *α*_M(IR),c_, the average angle between M(IR) and the polymer chain (c), for the PPO band at 1305 cm^-1^ (*α*_M(IR),c_ = 22.4°) and the PS band at 906 cm^-1^ (*α*_M(IR),c_ = 35°).^4^

*Raman – IR correlations for Raman tilt angle determination*

**Figure S2.** Correlations between the ⟨*P_2_*⟩_M,z_ (determined by Raman) and the corresponding ⟨*P_2_*⟩_c,z_ (determined by IR) for A) the 1305 cm^-1^ PPO band and B) the 623 cm^-1^ PS band. All values were determined from a series of stretched 50:50 PS/PPO films (type C samples). The slope of the linear fits, indicated directly on the graphs, corresponds to (3cos^2^(*α*_M,c_) – 1)/2, from which the Raman tilt angle *α*_M,c_ was determined: *α*_M,c_ = 33° for the 1305 cm^-1^ PPO band and *α*_M,c_ = 89° for the 623 cm^‑1^ PS band.

*Relative evaporation rate of the electrospinning solvents*

**Figure S3.** Evaporation behavior of the solvent systems illustrated by the normalized solvent quantity (NSQ) of A) pure CHCl_3_ and B) 50:50 CHCl_3_/ClBz mixture and by C) solvent volume fraction (SVF) of CHCl_3_ and ClBz in the 50:50 CHCl_3_/ClBz mixture as a function of time.

NSQs were determined by following the intensity of the 1215 cm^-1^ CHCl_3_ and of the 903 cm^-1^ ClBz bands using transflection IR spectroscopy. The NSQ values were determined by normalizing the intensity of the bands with their initial intensity. SVFs were determined by using the equation SVF_i_ = NSQ_i_ / (NSQ_CHCl3_ + NSQ_ClBz_), where *i* corresponds to either CHCl_3_ or ClBz. The time scale shown on the x axis corresponds to that of the film evaporation experiments and does not represent the actual evaporation time during the electrospinning process, which is faster by orders of magnitude. Nevertheless, we assume that the observed relative behaviors are comparable in both contexts, notably the changes in solvent composition along the jet path (shown in the inset).

The vertical hatched lines in panels A and B represent a crude approximation of when the remaining solvent quantity corresponds to the solvent mass fraction (values indicated directly on the graphs) required to achieve a *T*_g,eff_ of 25 °C in the corresponding electrospinning systems. Utilizing reported *T*_g_ values of 105.9 K (-167.3 °C) and 126.3 K (-146.9 °C) for pure CHCl_3_ and ClBz,^5^ respectively, and with the experimentally determined *T*_g_ of 483 K (210 °C) for PPO, the Fox model indicates in first approximation that this *T*_g,eff_ is attained when the PPO-CHCl_3_ system contains PPO and CHCl_3_ mass fractions of around 0.83 and 0.17 and the PPO-CHCl_3_-ClBz system contains PPO, CHCl_3_, and ClBz mass fractions of approximately 0.78, 0.005, and 0.215 (where the relative quantity of CHCl_3_ and ClBz respects their relation in Fig. S3B). According to the data of Figure S3, achieving this condition takes around 2.5 times longer when using the CHCl_3_/ClBz solvent mixture compared to pure CHCl_3_. While the quantitative value is only a rough estimation of the actual evaporation process, it is qualitatively consistent with the expectations and guides the understanding of the orientation and crystallization results.

**References**

1. S.C. Park, Y. Liang, H.S. Lee. “Quantitative Analysis Method for Three-Dimensional Orientation of PTT by Polarized FTIR-ATR Spectroscopy”. Macromolecules. 2004. 37(15): 5607-5614.

2. T. Lefèvre, C. Pellerin, M. Pézolet. “Characterization of Molecular Orientation”. In: J.M. Chalmers and R.J. Meier, editors. Comprehensive Analytical Chemistry. Elsevier, 2008. pp. 295-335.

3. M. Tanaka, R.J. Young. “Polarised Raman Spectroscopy for the Study of Molecular Orientation Distributions in Polymers”. Journal of Materials Science. 2006. 41(3): 963-991.

4. D. Lefebvre, B. Jasse, L. Monnerie. “Fourier Transform Infra-Red Study of Uniaxially Oriented Poly(2,6-dimethyl 1,4-phenylene oxide)-Atactic Polystyrene Blends”. Polymer. 1981. 22(12): 1616-1620.

5. C.A. Angell, J.M. Sare, E.J. Sare. “Glass Transition Temperatures for Simple Molecular Liquids and their Binary Solutions”. J. Phys. Chem. 1978. 82(24): 2622-2629.
